# Ultrasound-targeted microbubble destruction optimized HGF-overexpressing bone marrow stem cells to repair fibrotic liver in rats

**DOI:** 10.1186/s13287-020-01655-1

**Published:** 2020-04-03

**Authors:** Ting Sun, Hualin Li, Yun Bai, Min Bai, Feng Gao, Jie Yu, Rong Wu, Lianfang Du, Fan Li

**Affiliations:** 1 Department of Medical Ultrasound, Qingdao Municipal Hospital (Group), Qingdao, 266000 Shandong China; 2 Department of Medical Ultrasound, Shanghai General Hospital, Shanghai Jiao Tong University School of Medicine, 100 Haining Rd., Shanghai, 200080 China; 3 Department of Medical Ultrasound, Zibo Maternal and Child Health Hospital, Zibo, 255029 Shandong China

**Keywords:** Liver fibrosis, Liver injury, Bone marrow mesenchymal stem cells, Hepatic growth factor, Ultrasound, Microbubble, Stem cell transplantation

## Abstract

**Background/aims:**

Bone marrow mesenchymal stem cells (BMSCs) have shown their therapeutic potential in cytotherapy for liver fibrosis. However, the insufficient homing of BMSCs and undefined proliferation of BMSCs represent a significant challenge and largely limit the effective implementation. The aims of the present study were to determine whether stable expression of hepatic growth factor (HGF) in BMSCs coupled with ultrasound-targeted microbubble destruction (UTMD) technique could effectively and definitely alleviating carbon tetrachloride (CCl4)-induced liver fibrosis in rats.

**Materials and methods:**

A rat model of liver fibrosis was acquired by injection of carbon tetrachloride (CCl4). The experimental rats were randomly assigned to the four groups: normal, CCl4, BMSCs-HGF/US, and BMSCs-HGF/UTMD groups. The BMSCs, transfected by recombinant adeno-associated virus vector encoding human genome sequence of HGF (BMSCs-HGF), were transplanted in rat via the tail vein. The homing efficiency of BMSCs was observed by immunofluorescence staining. The liver function and its morphological changes were analyzed by biochemical tests and liver histology. The expression of liver fibrosis markers including α-smooth muscle actin (α-SMA), collagen I, and vimentin were examined by immunohistochemistry and quantitative real-time polymerase chain reaction.

**Results:**

The homing efficiency of BMSCs in the fibrotic liver was significantly greater with the application of UTMD. The biochemical markers of liver function and histopathological results showed significantly better improvement in BMSCs-HGF/UTMD group than the other groups, and the serum levels of biochemical markers returned to normal ranges in 12 weeks in this group. Furthermore, the expression levels of liver fibrosis markers (α-SMA, collagen I, and Vimentin) were all significantly lower in BMSCs-HGF/UTMD group in comparison with other groups.

**Conclusions:**

Our findings have demonstrated that stable expression of HGF in BMSCs and application of the UTMD technique facilitate the homing of BMSCs, and more importantly, which could further improve their alleviation of liver fibrosis. Therefore, these findings have an important clinical implication that AAV-BMSCs-HGF and UTMD hold promise as a novel therapeutic approach for liver fibrosis.

## Introduction

Liver fibrosis, if left untreated, can rapidly progress into the advanced stages and eventually liver failure [1]. Currently, liver transplantation is recognized as the most effective treatment strategy. Due to the limited number of the available liver donors, considerably high cost in a liver transplantation, and postoperative rejection, and better therapies are still in urgent need [2].

Recently, it has been a research breakthrough that stem cells are capable of regenerating and repairing damaged hepatocytes. In fact, bone marrow mesenchymal stem cells (BMSCs), a class of stem cells derived from the bone marrow with cross-system, trans-embryonic differentiation potential and high self-renewal ability have shown their therapeutic potential and advantages in cytotherapy for liver fibrosis caused by chronic liver diseases [3]. However, the insufficient homing of BMSCs to the target liver tissue appears to be a significant challenge, as the targeted homing efficiency is poor, and the ability to differentiate into ideal hepatocytes is much lower than that required quantity for liver repair [4]. The undefined proliferation of BMSCs in vivo was the key point which largely limits the effective implementation, which was also the controversial point of using BMSCs to repair the fibrotic liver.

Several previous studies have demonstrated that hepatic growth factor (HGF), a pleiotropic cytokine with a critical anti-fibrotic and anti-apoptotic role involved in the process of liver regeneration [5], induces the BMSCs migration via binding to c-met in the HFG-c-met axis. Migration of the bone marrow and cord blood mesenchymal stem cells in vitro is regulated by stromal-derived factor-1-CXCR4 and hepatocyte growth factor-c-met axes and involves matrix metalloproteinases. It has been reported that BMSCs in combination with HGF either by pretreating with HGF or injecting BMSCs and HGF had a better therapeutic effect than BMSCs alone [6]. However, these previous studies have limitations. First, exogenous HGF can be rapidly cleared from the target liver tissue, resulting in a short duration of the biological effect. BMSCs transfected with lentivirus or retrovirus overexpressing HGF have been used to maintain a stable and effective concentration of HGF, and another concern arises over this as these viral vectors have certain toxic effects to stem cells [7, 8]. In contrast to lentivirus or retrovirus, recombinant adeno-associated virus (rAAV) has more biosafety and lower immunogenicity, and its transduced gene can be stably expressed for longer time up to several months. Furthermore, previous research results of our team have shown that ultrasound-targeted microbubble destruction (UTMD) can promote a variety of gene vectors (plasmids, liposomes, rAAV, nanoparticles) into tumor cells and pluripotent stem cells, achieving attractive promising transfection efficiency [9]. Additionally, our previous study has found that the application of UTMD enhanced the homing of BMSCs to the acute injured liver, thereby producing better therapeutic effects [10].

In this study, we aimed at investigating whether stable expression of HGF in BMSCs coupled with the application of the UTMD technique could improve the capacity of BMSCs homing, stable differentiation of BMSCs, and whether the treatment could finally effectively alleviate liver fibrosis in rats.

## Materials and methods

### Cell culture

Rat BMSCs were provided by the Stem Cell Bank of the Chinese Academy of Sciences (CAS) (Shanghai, China). BMSCs were cultured in DMEM/F12 medium (Gibco, NY, USA) supplemented with 10% fetal bovine serum (FBS) (Gibco, NY, USA) and 1% penicillin-streptomycin solution (Gibco, NY, USA) in an incubator at 37 °C, 5% carbon dioxide (CO_2_). The cells were passaged at a ratio of 1:2 at the density reaching an approximately 80% confluence.

### Transfection of recombinant adeno-associated virus

A recombinant adeno-associated virus (rAAV) expression vector encoding human HGF (rAAV-HGF) genome sequence was used to stably express HGF and labeled with 4′,6-diamidino-2-phenylindole (DAPI) (Sangon Biotech, Shanghai, China). Prior to transfection, BMSCs were treated with rAAV-HGF for 6 h. Then, the cells were washed twice in phosphate buffer saline (PBS) (HyClone, UT, USA) and grown in DMEM/F12 cell culture medium for another 20 h. The efficiency of transfection was determined by the number of BMSCs expressing HGF, as measured by Western blotting, real-time quantitative reverse transcription–polymerase chain reaction (qRT-PCR), and immunofluorescence. The BMSCs transfected with rAAV-HGF were used in the subsequent transplantation for the cell therapy of liver fibrosis in rats.

### Western blotting analysis of HGF protein in transfected BMSCs

Western blotting analysis was conducted to examine the protein levels of human HGF in the total proteins isolated from rAAV-HGF-transfected BMSCs using RIPA buffer (Beyotime Biotechnology, Nanjing, China). A BCA protein assay kit (Beyotime Biotechnology, Nanjing, China) was used for quantification of the total proteins. Approximately 10 μg of the total proteins were separated on 10% SDS-PAGE gel (Beyotime Biotechnology, Nanjing, China) and transferred to a PVDF membrane (Bio-Rad, CA, USA). Membranes were blocked with 5% non-fat milk and followed by incubation with primary anti-HGF antibody (dilution of 1:1000) (Cell Signaling Technology, MA, USA) at 4 °C overnight. The membranes were then incubated with the secondary antibodies, horseradish peroxidase (HRP)-conjugated goat anti-rabbit IgG (1:2000) (Beyotime Biotechnology, Nanjing, China) at RT for 1.5 h. The membranes were washed in the TBST solution for three times with each 10 min. The immunoblots were visualized using the chemiluminescence imaging system (Bio-Rad, CA, USA) according to the manufacturer’s protocol. ImageJ software (Bio-Rad, CA, USA) was used to analyze the protein levels of HGF which were normalized to β-actin.

### Animal model of liver fibrosis

Male Sprague Dawley (SD) rats aged 6 weeks were provided by the Animal Center of the Shanghai General Hospital (Shanghai, China), and they were housed in an air-conditioned room under sterile conditions. For a rat model of liver fibrosis, SD rats were fed with 5% ethanol and were subcutaneously injected with 40% carbon tetrachloride (CCl4) (Sigma-Aldrich, MO, USA) diluted in olive oil at a 1:1 (v/v) ratio (0.5 ml/kg) three times per week for consecutive 9 weeks. Rats as control were administered with a subcutaneous injection of olive oil alone three times a week for a total of 9 weeks. After completion of the treatment, CCl4-induced liver fibrosis in SD rats was evaluated and confirmed by biomedical examinations of serum biomarkers for liver damage as well as pathological analysis of liver tissues.

All the study protocols involving animal experiments were reviewed and approved by the Ethics Committee of the Shanghai General Hospital (Shanghai, China).

### The transplantation BMSC-HGF in rats

As the limited efficiency of BMSCs alone in the injured liver and unstable effect of exogenous HGF on fibrotic liver demonstrated by previous researches [10, 11], we did not include separate BMSCs and HGF group in this study. Twelve normal rats received BMSCs-HGF transplantation were used as a control group. Thirty-six liver fibrosis rats were randomly assigned into the three groups: (1) CCl4 group (*n* = 12), rats received BMSCs-HGF transplantation, (2) BMSCs-HGF + ultrasound (US) group (*n* = 12) in which rats received BMSCs-HGF transplantation and US therapy, and (3) BMSCs-HGF/UTMD group (*n* = 12) in which rats were treated with BMSCs-HGF transplantation and UTMD. The US and UTMD procedures were performed as described previously [10]. The position of the liver was determined using a diagnostic ultrasound system Sequoia 512 (SIEMENS, Germany). The body surface marker was made when the second hepatic hilum was displayed, then the center of the ultrasonic therapeutic apparatus (Physioson Elektromedizin AG, Germany) probe was placed. Specifically, the probe area is 5 cm^2^, the irradiation parameter is 1:5 duty ratio, the ultrasound intensity is 1.5 W/cm^2^, and the irradiation time is 10 min.

In the BMSCs-HGF/US group, rats were given US irradiation prior to the stem cells transplantation, BMSCs-HGF (approximately 2 × 10^6^ cells) were subsequently injected through the tail vein. In the BMSCs-HGF/UTMD group, rats were treated with UTMD with the injection of 300 μL SonoVue suspension and US irradiation, following which BMSCs-HGF (approximately 2 × 10^6^ cells) were infused in the rats. All the experimental rats were observed for 2 months and assessed for the therapeutic effects every 2 weeks.

### Immunofluorescence staining of BMSCs’ homing in the rat liver

Liver-frozen sections were cut into 4–10 μm and then observed under a fluorescence microscope. Quantified and analyzed the DAPI-labeled cells represented the number of homing BMSCs that were analyzed.

After BMSCs were transfected with human HGF-encoded rAAV, double immunofluorescence staining of human HGF and fibrous actin (F-actin) was performed to evaluate the long-term effect of the transplanted BMSCs on HGF expression at the cellular level, which was measured by the fluorescence intensity of HGF normalized to that of F-actin. As F-actin, a cytoskeletal protein was consistently expressed in BMSCs, immunofluorescence staining of F-actin was used for normalization to the cell number. First, liver-frozen sections were immersed in 0.3% Triton X-100 (Sangon Biotech, Shanghai, China) for increasing cell membrane permeability. Then the slices were blocked with 3% bovine serum albumin (BSA) (Cell Signaling, Danvers, MA, USA) at room temperature (RT) for 10–15 min. Slices were then incubated with the anti-human-HGF antibody (1:200, ABCAM, Cambridge, MA, USA) at RT for 2 h and washed three times with PBS. After that, slices were incubated with rhodamine-labeled phalloidin (Thermo Fisher, Waltham, MA, USA) and goat anti-rabbit IgG secondary antibody (Alexa Fluor 488, 1:1000, Thermo Fisher) for 30 min. Images were acquired under a confocal laser scanning microscope (Zeiss, Oberkochen, Germany). The relative fluorescence intensity in each field was acquired by ImageJ software.

### Biochemical tests for liver function

Serum alanine aminotransferase (ALT), aspartate aminotransferase (AST), alkaline phosphatase (ALP), total protein (TP), and albumin (ALB) were determined following standard protocols using an automatic biochemical analyzer (Beckman Coulter, CA, USA).

### Histological examinations of liver tissue

Rat liver tissues were excised and fixed in 4% neutral formalin solution for 48 h. Then the tissues were embedded, dehydrated, cleared, and cut into 4-μm sections. The sections were either stained with hematoxylin and eosin (H&E) for evaluation of hepatic necrosis and recovery or stained with Van Gieson (Sigma-Aldrich, MO, USA) for visualization of collagen fibers, and analyzed under a light microscope using standard protocols.

### Immunohistochemistry analysis of liver fibrosis markers

Immunohistochemistry analysis of fibrosis markers, including α-smooth muscle actin (α-SMA), vimentin, and collagen I, was performed to assess the degree of liver fibrosis in rats. In brief, the liver tissue slices of the SD rats were dewaxed, immersed, and incubated in 3% hydrogen peroxide (H_2_O_2_) (Sangon Biotech, Shanghai, China) at RT for 10 min. The slices were then blocked with BSA (Cell Signaling, Danvers, MA, USA). Anti-α-SMA antibody (Cell Signaling Technology, MA, USA) (1:100), anti-vimentin antibody (Abcam, MA, USA) (1:100), and anti-collagen I (Cell Signaling Technology, MA, USA) (1:100) were added to the slides and incubated at 4 °C overnight. Next, the slices were incubated with goat anti-rabbit IgG secondary antibody (Beyotime Biotechnology, Nanjing, China) at RT. 3,3′-Diaminoben-zidine (DAB) was used as a developing reagent for visualizing expression levels of the fibrosis markers. The degree of liver fibrosis was evaluated under a microscope.

### Quantitative RT-PCR analysis of liver fibrosis markers’ expression

The mRNA levels of liver fibrosis markers: α-SMA, collagen I, and vimentin were quantified by qRT-PCR analysis. Briefly, total RNA was extracted from the rat liver tissues using Trizol Reagent (Invitrogen, Grand Island, NY, USA) in accordance with the manufacturer’s manual. Prime-Script RT Master Mix reagent kit (Takara, Dalian, China) was used for the synthesis of complementary DNA (cDNA) according to the manufacturer’s instructions. Real-time qRT-PCR reactions were prepared in a total volume of 20 μL using Premix Ex reagent kit (Takara, Dalian, China) and performed on an automated ABI ViiATM 7 real-time PCR system (Applied Biosystems, Waltham, MA, USA). The real-time PCR reactions of all samples were performed in triplicate, and the resulting data were calculated by 2^**-ΔΔCt**^ algorithm. The mRNA levels of α-SMA, collagen I, and vimentin were normalized to GAPDH.

**Table:** Sequences of primers used for qRT-PCR (rat)

Gene	Forward sequence	Reverse sequence
α-SMA	GCGTGGCTATTCCTTCGTTA	ATGAAGGATGGCTGGAACAG
Collagen I	GCCTCCCAGAACATCACCTA	ATGTCTGTCTTGCCCCAGT
Vimentin	CTGCTGGAAGGGGAGGAGAG	GGTCATCGTGGTGCTGAGAAGC
HGF	CAATCCAGAGGTACGCTACGAC	GTGCCTGATTCTGTGTGATCC
GAPDH	TGCACCACCAACTGCTTAG	GATGCAGGGATGATGTTC

### Statistical analysis

Statistical analysis was performed using SPSS 20.0 software (Chicago, IL, USA). All the values were expressed as mean ± standard deviation (SD). A comparison between groups was made in the way of paired Student’s *t* tests. Results with *p* values less than 0.05 were considered statistically significant.

## Results

### Expression of HGF in BMSCs

**Fig. 1:**
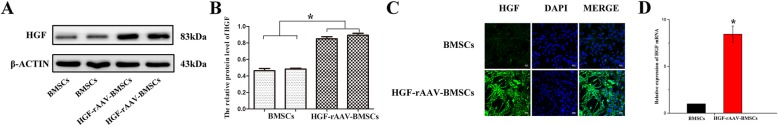
Transfection and overexpression of the HGF gene in BMSCs. The expression vector rAAV-HGF labeled with DAPI was introduced into the BMSCs. **a** Western blot analysis of HGF protein in BMSCs after transfection (using BMSCs as control). **b** The relative quantification analysis of HGF protein using ImageJ software(*p < 0.05). **c** Immunofluorescence of HGF in BMSCs under confocal microscope (× 400). **d** Real-time PCR analysis of HGF mRNA in transfected BMSCs (using BMSCs as control) (*p < 0.05)

To enhance the homing capacity of BMSCs, the expression vector rAAV-HGF was introduced into the cells. Following the rAAV-HGF transduction, the expression of human HGF gene at the RNA, protein, and cellular levels was determined by real-time qRT-PCR, Western blotting, and laser scanning confocal microscopy (Fig. 1a–d). Figure 1a, b demonstrated the high expression of HGF after transfection, indicating high transfection efficiency. Furthermore, the amount of green fluorescence (HGF protein) under the laser confocal microscope (Fig. 1c) was consistent with the WB result. Figure 1d showed that HGF mRNA and protein levels were significantly increased in the BMSCs 6 h after rAAV-HGF vector was transfected in comparison with the control BMSCs. These data indicated that the expression of HGF was successfully achieved in the transfected BMSCs.

### Validation of a rat model for liver fibrosis

**Fig. 2:**
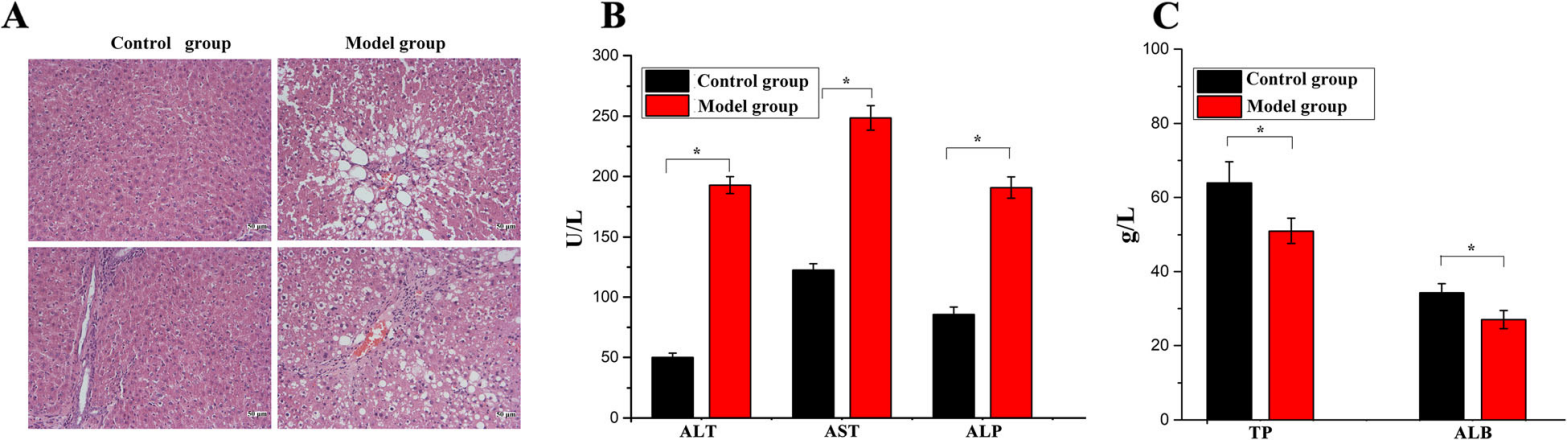
Establishment and validation of liver fibrosis induced by CCI4 in rats. **a** Hematoxylin and eosin staining (H&E staining) in the model group, showing the absence of normal hepatic lobule, instead of collagen fibers. And the portal tracts were thickened. The hepatic histologic structures were normal in the control group (normal rats) (× 200). **b**, **c** The changes of important serological markers in the control and model group (*p < 0.05)

Liver fibrosis in rats was successfully induced 9 weeks after the intraperitoneal injection of CCl4 as evaluated by histopathological examinations of the liver tissues (Fig. 2). As shown in the figure, H&E staining of the liver tissues showed an absence of normal hepatic lobule structure and presence of a large amount of collagen fibers under a microscope (Fig. 2a). In addition, portal tracts were thickened and conjected with chronic inflammatory cells along the fibrous septa (Fig. 2a). Furthermore, the changes in serum biomarkers suggested the decline of liver function (Fig. 2b, c). These pathological and biochemical changes of the liver tissues in the rats indicated the establishment of the CCl4-induced rat model for liver fibrosis.

### The homing efficiency of BMSCs in the rat liver

**Fig. 3:**
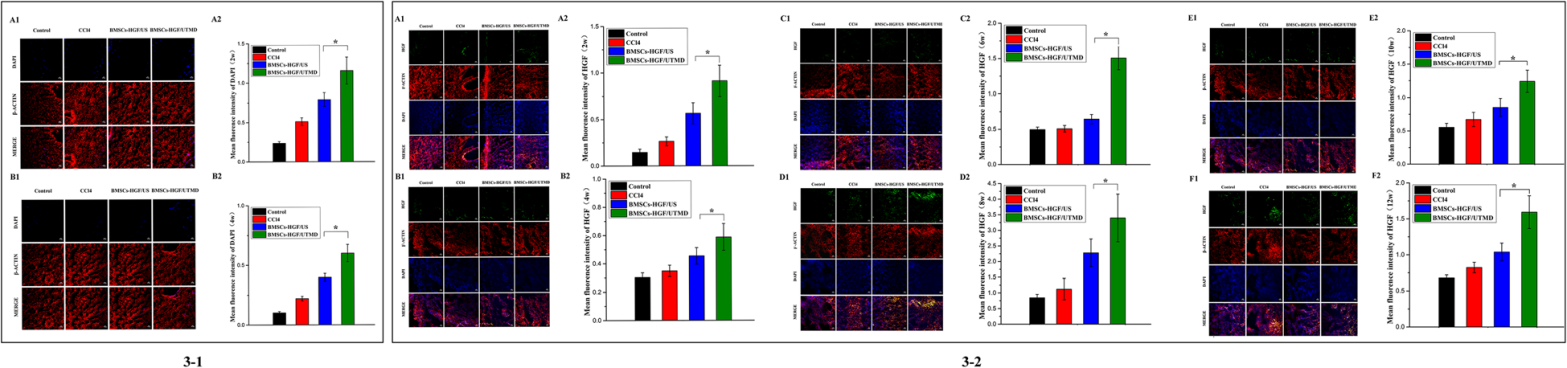
The homing efficiency of BMSCs into the liver. (3-1 A1, B1) The number of BMSCs reaching the liver was observed under a fluorescence microscope at 2 weeks and 4 weeks (× 200). (3-1 A2, B2) The mean fluorescence intensity was displayed for quantification of the images (*p < 0.05). (3-2 A1, B1, C1, D1, E1, F1) Human HGF and F-actin staining to analyze the homing rate of BMSCs at 2 weeks, 4 weeks, 6 weeks, 8 weeks, 10 weeks, and 12 weeks (× 200). (3-2 A2, B2, C2, D2, E2, F2) The mean fluorescence intensity of HGF was calculated for quantification of the BMSCs (*p < 0.05)

After injection of BMSCs-HGF via tail vein, DAPI-labeled BMSCs with blue fluorescence were observed in the liver portal area at 2 weeks and 4 weeks. In comparison with the control and BMSCs-HGF/US group, the mean fluorescence intensity was much higher in BMSCs-HGF/UTMD group (*p* < 0.05) (Figs. 1, 2, and 3).

The double immunofluorescence staining of human HGF and F-actin showed that the green fluorescence representing the consistent expression of human HGF was greatly stronger in BMSCs-HGF/UTMD group than the other groups in 12 weeks, which demonstrated the increased BMSCs homing effect could be maintained for a long time (Figs. 3-2 E1, E2, F1, F2).

### The recovery of liver injury in rat models

**Fig. 4:**
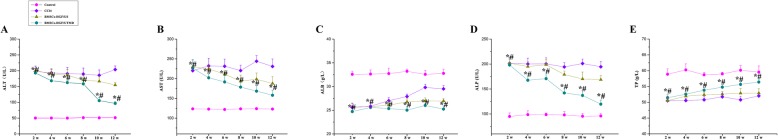
Effects of the BMSCs on the serum levels of biochemical tests for the liver function. At time point of 2 weeks, 4 weeks, 6 weeks, 8 weeks, 10 weeks, and 12 weeks after BMSCs treatment, serum levels of ALT (**a**), AST (**b**), ALB (**c**), ALP (**d**), and TP (**e**) of the four groups were analyzed and compared. Experiments were conducted in three batches (*p < 0.05). The data demonstrated the values of biochemical tests for the liver function were significantly improved in the BMSCs-HGF/UTMD group at different point times (the decrease of ALT, AST, and ALP and the increase of ALB and TP). (*p < 0.05, compared with the other three groups at the same time; *# < 0.05, made self-contrast at different points)

As shown in Fig. 4, the quantitative values of liver function markers (ALT, AST, ALP, TP, and ALB) were significantly improved (*p < 0.05) in BMSCs-HGF/UTMD group compared to CCl4 group at 2 weeks. Moreover, in comparison with BMSCs-HGF/US group, BMSCs-HGF/UTMD yielded a stronger recovery effect over time. Notably, the serum levels of those biochemical markers returned to the normal ranges at 12 weeks (Fig. 4).

**Fig. 5:**
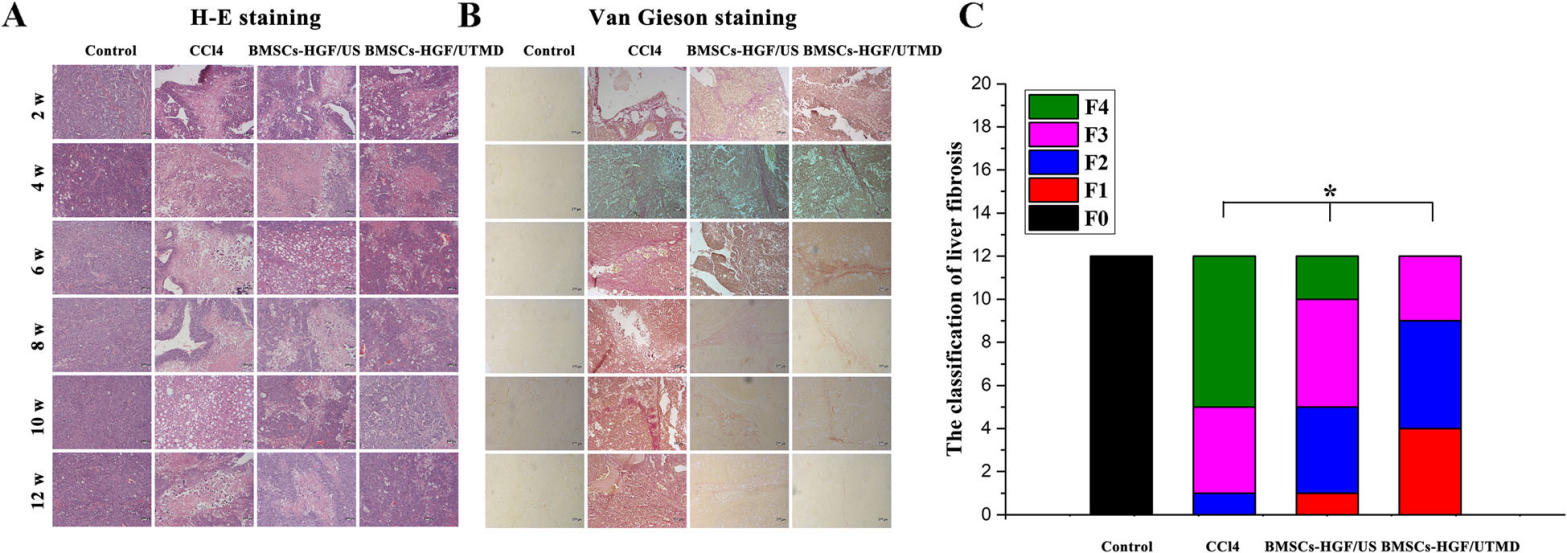
H-E staining and Van Gieson staining demonstrated the histological changes of liver structures. H-E and Van Gieson staining of liver section was made at time point of 2 weeks, 4 weeks, 6 weeks, 8 weeks, 10 weeks, and 12 weeks after injection of BMSCs, respectively (× 200). **a** H-E staining of the liver which reflected the hepatic structures, **b** on the right was collagen fiber staining, and **c** a stacked map histogram on the right that comprehensively analyzes the stages of fibrosis in each group of rats (refer to METAVIR period standard, divided into F0~F4)

Liver histology was performed every 2 weeks using H&E and Van Gieson staining to evaluate the recovery of liver injury in rats. As illustrated in Fig. 5, the CCl4 group showed CCl4-induced persistent toxicity with histological changes, including extensive ballooning hepatocytes and diffuse steatosis and necrosis. After BMSCs transplantation, hepatic cords, necrotic cells, and inflammatory cells were reduced over time, and the histological changes towards the recovery of liver injury were the greatest in the BMSCs-HGF/UTMD group. Moreover, necrotic areas were significantly reduced at 6 weeks after treatment in the BMSCs-HGF/UTMD group. As hepatocytes regenerated, at 10 weeks following treatment, a large number of reconstituted normal liver histological features were observed in the BMSCs-HGF/UTMD group. In contrast to the BMSCs-HGF/US group, the BMSCs-HGF/UTMD group showed better repairing abilities.

Referring to METAVIR points’ period standard, the F0–F4 level was divided to indicate liver fibrosis stages. We could see that the proportion of rats in stage F4 decreased to 0, and the number of rats in stages F2, F3, and F1 increased in BMSCs-HGF/UTMD. However, there was still no F1 grade rat in BMSCs-HGF/US group, although the F4 ratio was decreasing. That is, the effect of anti-fibrosis was the most significant in BMSCs-HGF/UTMD group.

### The fibrosis condition in rat models

As shown in Fig. 5, severe diffuse collagen deposition particularly around portal triad occurred in the CCl4 group. It was noticed that the amount of collagen fibers was significantly decreased after 4 weeks in BMSCs-HGF/UTMD group compared to the CCl4 group. Further, significant better therapeutic effects were achieved in BMSCs-HGF/UTMD group than in BMSCs-HGF/US group (*p* < 0.05).

**Fig. 6:**
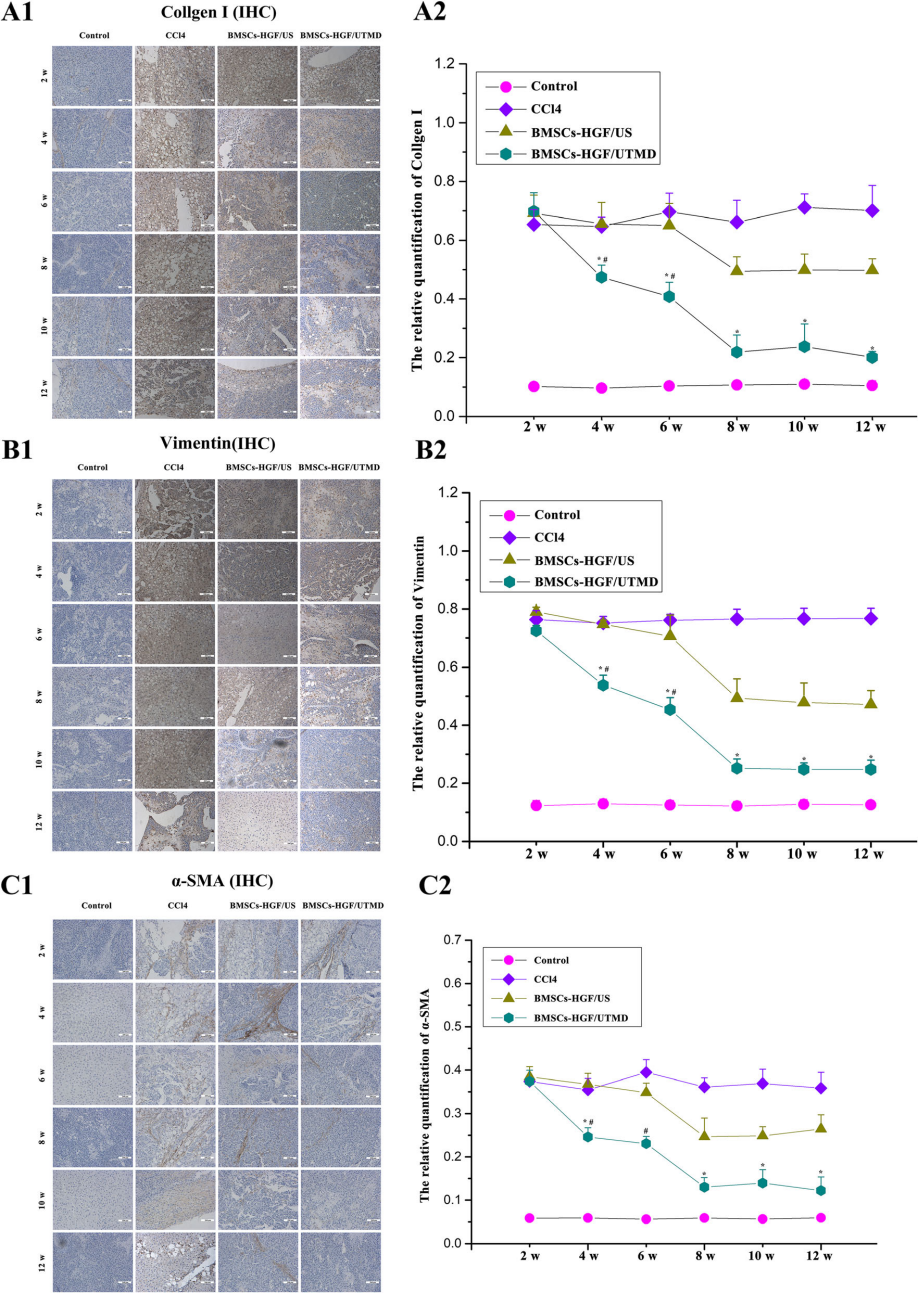
Immunohistochemical staining analysis using collagen I and vimentin and the relative quantitative curve using ImagePro plus software. (A1) Immunohistochemcial staining for collagen I in the four groups at each detection time(× 200), (B1) immunohistochemcial staining for vimentin in the four groups (× 200), (C) immunohistochemcial staining for α-SMA(× 200), and (A2, B2, C2) the relative quantification of immunohistochemistry collagen I, α-SMA, and vimentin. As was shown in the figure, the amount of collagen I, α-SMA, and vimentin were significantly decreased by the BMSCs treatment after 4 weeks in BMSCs-HGF/UTMD group. Also, the presence of fibers gradually decreased to a relatively low level after 8 weeks. (*p < 0.05, compared with the other three groups at the same time; *# < 0.05, made self-contrast at different points)

**Fig. 7:**
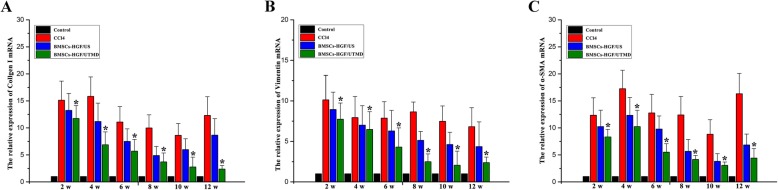
The expression of a-SMA, collagen I, and vimentin mRNA. The a-SMA, collagen I, and vimentin mRNA levels were analyzed by qRT-PCR. Results were in consistent with Fig. 6. The mRNA of the three fibrotic indices was the lowest in BMSCs-HGF/UTMD group (mRNA in the control group was used as a benchmark) (*p < 0.05)

As shown in Fig. 6, there were a large proportion of the positive α-SMA cells in the liver portal area in the CCl4 group. After BMSCs transplantation, the α-SMA-positive area and the fibrotic structure were gradually decreased over time both in US and UTMD groups; however, the reduction of that fibrosis was even faster in BMSCs-HGF/UTMD group. A similar trend was found in collagen I and vimentin expression. The collagen fibers in the liver architecture of BMSCs-HGF/UTMD were reduced to nearly normal levels at a 10-week point. The mRNA levels of α-SMA, collagen I, and vimentin mRNA levels analyzed by qRT-PCR further verify the difference among the groups (Fig. 7).

## Discussion

In this study, we investigated the stable expression of HGF by BMSCs transplantation with UTMD approach and their effects in suppressing liver fibrosis and promoting liver regeneration in rats with CCl4-induced liver fibrosis. The novel findings of this study were summarized as follows: (1) the homing efficiency of BMSCs to the injured liver was significantly improved by stable expression of HGF in BMSCs with UTMD; (2) following treatment with BMSCs-HGF and UTMD, the liver function and morphology of fibrotic liver were significantly improved, and the serum levels of biochemical markers returned to normal ranges 12 weeks after treatment; and (3) liver fibrosis was significantly alleviated following treatment of BMSCs-HGF and UTMD.

In this study, CCl4 was used to induce liver fibrosis in rats, as it has been thought that CCl4-induced liver fibrosis in animal models has presented similar characteristics to those features in patient’s chronic liver disease [12]. In CCl4-induced hepatotoxicity, CCl4 activates a large number of free radicals by cytochrome P4502E1, leading to lipid peroxidation, DNA damage, and cell death [13]. In the study, serum biomarkers of ALT, AST, ALP, ALB, and TP were significantly elevated after CCl4 treatment in rats, which was accompanied by the production of collagen fibers soon afterwards. The increases in serum levels of ALT, AST, ALP, ALB, and TP were indicative of liver injury, which was in accordance with previous findings.

Chronic liver disease can progress to liver fibrosis, cirrhosis, liver cancer, and eventually end-stage liver failure that usually need liver transplantation. The current treatment options for liver fibrosis and cirrhosis are very limited [14]. Alternative therapeutic approaches have been proposed, such as cell-based treatment. The therapeutic effectiveness of BMSCs for the liver fibrosis has been extensively reported [15, 16]. Previous studies in animal models have indicated that BMSCs and the secreted factors have the ability to ameliorate liver fibrosis and stimulate the regeneration of hepatocytes [17].

HGF is an important factor and plays a role in promoting hepatocyte regeneration and anti-fibrosis. Previous studies have demonstrated HGF has potent cytoprotective effects on liver cirrhosis [18]. Unfortunately, the exogenous HGF is extremely unstable, which could not maintain sustainable high levels in blood circulation, even if multiple and repeated injections were administrated [19]. Therefore, lentivirus- or adeno-associated virus has been widely used for stable and prolonged expression of HGF in vivo. Previous studies have shown that BMSCs overexpressing HGF could significantly enhance hepatocyte regeneration, inhibit apoptosis, and reverse the progression of liver fibrosis [20, 21]. UTMD was proved to be an effective method to deliver genes and drugs by acoustic cavitation effects generated by the interaction of ultrasound and microbubbles [22, 23]. Moreover, UTMD has been shown to promote targeted homing of BMSCs in different organs [24, 25].

Based on previous findings, we explored the effects of UTMD combined with BMSCs overexpressing HGF in rat models of liver fibrosis. Expectedly, the migration of BMSCs into the liver was significantly enhanced in BMSCs-HGF/UTMD group, demonstrated by the fluorescence intensity of DAPI. It was of note that the fluorescence of HGF could be maintained 10–12 weeks after cell transplantation, which illustrated the long-term effect of increased homing of BMSCs expressing HGF. In addition, the recovery of liver function in the BMSCs-HGF/UTMD group occurred earlier than the other groups, accompanied by further decreasing of the expression levels of liver function markers in BMSCs-HGF/UTMD group than the other groups.

It may merit the attention that histopathologic examinations revealed that the therapeutic effectiveness of BMSCs-HGF/UTMD in amelioration of liver fibrosis and restoration of liver architecture. H-E staining confirmed that UTMD and BMSCs promoted the repair of the injured liver. Accordingly, immunohistochemistry showed that BMSCs had an anti-fibrotic effect, as evidenced by decreasing expression of α-SMA, vimentin, and collagen I. There was significantly statistically difference in the improvement of rat fibrosis between BMSCs-HGF/UTMD and BMSCs-HGF/US group, and this was likely attributed to a synergistic effect of ultrasound microbubbles rather than ultrasound alone.

As was shown in the literature, BMSCs exert a protective effect against liver fibrosis [26, 27]. The exact underlying mechanisms may be the following: BMSCs and its secretion may inhibit the activation and proliferation of hepatic stellate cells, and BMSCs inhibit apoptotic process of hepatocytes and promote hepatocyte proliferation [14]. Besides, HGF has the potential to upregulate the production of matrix metalloproteinase (MMP) and thus induce anti-fibrotic effect [28]. Thus, we proposed that the transplantation HGF-rAAV-BMSCs after UTMD pre-irradiation liver could enhance anti-fibrosis effect. In addition to UTMD that enhanced the homing of BMSCs into the liver, it can be speculated that the stable differentiation of BMSCs into hepatocytes in vivo after injection was also an important point.

Our study had some limitations. One was that the specific cytokines and molecular pathway underlying the anti-fibrotic effect of BMSCs have not been elaborated. Another was lacking the direct demonstration of BMSCs in a long-term living. Further in-depth studies are underway in our laboratory.

In conclusion, our results have indicated that the stable expression of HGF was achieved by transplantation of rAAV-BMSCs-HGF coupled with the UTMD technique, which facilitated the homing of BMSCs to the injured liver and thereby improved the alleviation of liver fibrosis in rats. As such, rAAV-BMSCs-HGF and UTMD could be served as a novel therapeutic approach for liver fibrosis. Further studies are needed to assess the application of UTMD technique in the treatment of patients with liver fibrosis for stem cell therapy.

## Data Availability

The data sets analyzed during this study are available from the corresponding author on reasonable request.

## Abbreviations

rAAV: Recombinant adeno-associated virus
DAPI: 2-(4-Amidinophenyl)-6-indole-carbamidine
PBS: Phosphate-buffered saline
RIPA: Protein lysis buffer radioimmunoprecipitation assay
BCA: Bicinchoninic acid
PMSF: Phenylmethanesulfonyl fluoride
TBST: Tris-buffered saline with Tween20
